# Efficient low temperature Monte Carlo sampling using quantum annealing

**DOI:** 10.1038/s41598-023-33828-2

**Published:** 2023-04-25

**Authors:** Roland Sandt, Robert Spatschek

**Affiliations:** 1 Structure and Function of Materials, Institute of Energy and Climate Research, Forschungszentrum Jülich GmbH, 52425 Jülich, Germany; 2 JARA-ENERGY, 52425 Jülich, Germany

**Keywords:** Statistics, Computational methods, Statistical physics

## Abstract

Quantum annealing is an efficient technology to determine ground state configurations of discrete binary optimization problems, described through Ising Hamiltonians. Here we show that—at very low computational cost—finite temperature properties can be calculated. The approach is most efficient at low temperatures, where conventional approaches like Metropolis Monte Carlo sampling suffer from high rejection rates and therefore large statistical noise. To demonstrate the general approach, we apply it to spin glasses and Ising chains.

## Introduction

The recent advent of quantum annealing (QA) is an important step towards the development of quantum computing in the future, which will significantly boost also statistical physics and materials science modeling. In general, QA, as implemented by the company D-Wave, allows to find efficiently ground state configurations of discrete optimization problems, with many possible applications in academia and industry^1–5^. There are many problem types to which QA has been applied, like the demonstration of scaling or algorithmic advantages for QA in specific problem classes^6–8^. So far, applications of QA in the field of materials science are still rare, and among them are the determination of equilibrium microstructures with long-range elastic interactions^9^, phase transitions in the transverse field Ising model^10^, the investigation of energy states of frustrated magnetic systems via the Shastry-Sutherland model^11^ and the designing of metamaterials^12^. Another example is the combined use of quantum annealers and Boltzmann machines to sample spin glasses and to predict molecular dynamics data of a $\mathrm {MoS_2}$ layer^13^.

The concept of QA is to initialize the system’s Hamiltonian at cryogenic temperatures in a well defined ground state, and then to smoothly convert the energy landscape such that it represents the desired optimization problem^14,15^. If this adiabatic transformation is performed carefully, the system ends up in the ground state of the destination Hamiltonian. An explicit finite temperature modeling of this transition has been performed for the Sherrington-Kirkpatrick spin glass model, see^16,17^ and references therein. However, apart from the stochastic nature of the approach itself, the preparation, transformation and readout process are not perfectly adiabatic, noise-free and decoupled from the environment, hence frequently states with higher energy are found, especially for Hamiltonians with small energy gaps. For a typical QA experiment, multiple repetitions and reads are used to determine the true ground state. In this paper we demonstrate that this deficit of the technology can actually be turned into a virtue, as it allows to determine finite temperature thermodynamic properties extremely efficiently. Related to that, the concept of using QA as (noisy) Gibbs sampler has been discussed recently^18,19^, but it turns out that a tuning of the temperature for performing quantitative simulations is challenging. Moreover, it has been shown that at least for some machine architectures degenerate ground states are sampled unequally with an exponential bias, contrary to the thermodynamic equilibrium concept that equal energy states should be visited with the same probability in the canonical ensemble, therefore demanding special attention^20–24^.

From a materials science perspective, the ground state configuration at temperature $T=0\,\textrm{K}$ is often only of limited interest for many practical applications. For example, for a ferromagnet, all spins are aligned in the ground state, whereas for finite temperatures thermal fluctuations lead to finite correlation lengths, phase transitions and temperature dependent magnetizations. A conventional approach for a statistical modeling of such properties is to use Monte Carlo (MC) sampling techniques, as an explicit computation of the partition function is typically not feasible due to the vast size of the phase space. The probably most prominent approach for such computations is the generation of discrete Markov chains using Metropolis transition probabilities, which generate a sequence of configurations which obey Boltzmann statistics, and therefore allow to express the ensemble average through the easier calculation of time averages along these Markov chains^25,26^. In practise, a transition from one state to another is taking place with probabilities depending on the energy difference $\Delta E$ between two configurations according to a Boltzmann distribution $p\sim \exp (-\beta \Delta E)$ with $\beta = 1/k T$ with the Boltzmann constant *k*. Usually, such approaches are inefficient at low temperatures, as then the rejection rate for new configurations is very high, and hence an insufficient sampling of the phase space is achieved with trapping in local minima, resulting in noisy predictions of the desired thermodynamic properties. Another important sampling strategy was developed by Wang and Landau, using a non-Markovian algorithm to extract the density of states via a flat histogram technique, from which all desired thermodynamic properties can be calculated^27^. Besides these major techniques, Dall et al. developed an algorithm to sample the Boltzmann distribution fast at low temperatures. However, this algorithm is most suitable for systems with short range interactions^28^. Another possibility for the fair sampling of ground and degenerate states is the introduction of parallel tempering with isoenergetic cluster updates in Monte Carlo methods^29^ or the combination with simulated annealing on a quantum annealer^30,31^. We mention that Boltzmann machines, which can serve as a link between machine learning and statistical thermodynamics^32^, are investigated in the context of QA^33–36^ from a computer science perspective, but to the best of our knowledge, the direct application of QA for classical finite temperature modeling for statistical physics and materials science has not yet been accomplished and is the subject of the present paper.

## Results

### Spin glass

**Figure 1:**
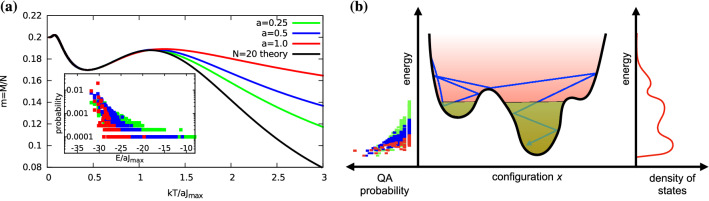
Mean magnetization and probability distribution of a spin glas and sampling strategy. (**a**) The plot shows the temperature dependent magnetization of an $N=20$ random coupling spin glass. The QA sampled values coincide with the theoretical results in the low temperature regime, whereas for elevated temperatures the energy rescaling factor *a* affects the quality of the results. The probability for getting a state *x*, which is estimated using 10,000 repetitions of the sampling, depends to good approximation only on its energy *E*(*x*) and follows essentially a Boltzmann distribution, as shown as inset. Different rescaling factors $a>0$ shift the distributions to higher or lower energies. (**b**) Illustration of the different sampling strategies. The blue trajectory illustrates the Markov chain generated by the Metropolis algorithm to generate a Boltzmann probability distribution (red shading). Alternatively, the Wang-Landau approach constructs the density of states, from which thermodynamic properties can be predicted. In contrast, the QA approach identifies low energy configurations (green shading), which are taken as most representative fraction of the phase space for low temperature expectation values.

The key feature of the quantum annealer is that it finds preferentially configurations which are close to the global energy minimum of the phase space. As a first illustration how to determine the low temperature thermodynamics from these configurations, we use a spin glass^37,38^ with random couplings, which is given by the Hamiltonian

1 $$\begin{aligned} H = \sum _{i<j} J_{ij} s_i s_j + \sum _i h_i s_i \end{aligned}$$

with $N=20$ spins $s_i=\pm 1$ and random values for the coupling constants, $J_{ij}, h_i\in [-J_{\textrm{max}},J_{\textrm{max}}]$, $J_{\textrm{max}}=1/2$. As the matrix $J_{ij}$ is fully populated, the model also includes long-range interactions. We point out that due to the random couplings, the energy landscape of the spin glass contains many states with nearby energy values without degeneracy, which avoids the issue of potentially unfair sampling of isoenergetic states. An example from materials science for such a spin glass are misfitting coherent grains in a polycrystalline solid, where the coupling constants result from elastic long-range interactions and external forces^9^. We repeat the quantum annealing read out process 10,000 times to get an estimate of the distribution of identified states, as due to the above mentioned reasons also higher energy states are found in practise. Therefore, we obtain a (sub-)set of states $S = \{x_i\}$, and each configuration consists of the value of spin variables, $x_i = (s_1^{(i)}, \ldots , s_N^{(i)})$, for which the resulting probability distribution is illustrated in the inset of Fig. 1a.

The distribution of the states depends to good approximation only on the energy of the individual configurations and follows (roughly) a Boltzmann distribution (with different effective temperatures), as has been discussed in the literature^18,19^, although it should be noted that quantum fluctuations can lead to deviations from the purely thermal probability distribution^39^. For the following steps it is important to mention that the explicit form of the distribution is not critical, and we only exploit the fact that states with low energies are found preferentially.

Additionally, a rescaling of the Hamiltonian $H\rightarrow aH$ by a factor $0<a<1$ allows to sample regions of the phase space with higher energy (see inset in Fig. 1a), requiring to switch off the automatic rescaling of the coupling constants by the D-Wave framework. The smaller *a* is chosen, the more high energy configurations are sampled. Explicitly, for the $N=20$ spin glass with a configuration space of size $2^N\approx 10^6$, we use 10,000 reads, which lead to around 300 (for $a=1.0$) to 5,000 (for $a=0.25$) distinct configurations in the subset *S* (the actual numbers fluctuate due to the non-deterministic behavior). We note that the concept of the rescaling of coupling constants has been previously used to effectively change the temperature of the distribution generated by the quantum annealer^18,19^, which is however not required here.

To obtain a numerical estimate of the canonical partition function using QA, we take the identified distinct low energy configuration set *S* and use the approximated canonical partition function

2 $$\begin{aligned} Z_a = \sum _{x\in S} \exp (-\beta H(x)), \end{aligned}$$

which obviously becomes more accurate for a better sampling of the low energy configurations. Notice that the desired (and given) inverse temperature $\beta$ is typically not related to the effective one related to the probability distributions of the QA sampling. With the estimated Boltzmann probability $p_a(x)= \exp (-\beta H(x))/Z_a$ of a state *x* we can obtain estimated expectation values of an observable *A*(*x*) according to

3 $$\begin{aligned} \langle A\rangle = \sum _{x\in S} p_a(x) A(x). \end{aligned}$$

We emphasize that the set *S* is significantly smaller than the size $2^N$ of the phase space, and therefore estimated values can be calculated efficiently also for large values of *N*, for which a direct computation of the partition function is no longer feasible. Furthermore, the same set is used for all temperatures, and therefore it is not necessary to create new chains of configurations like for Metropolis sampling. In this sense, the proposed algorithm is comparable to multicanonical approaches employing Wang-Landau sampling. As *S* contains mainly the low energy configurations, we expect that the estimated expectation values get accurate for low temperatures, i.e. large values of the inverse temperature $\beta =1/kT$.

This expectation is confirmed in Fig. 1a for the magnetization per spin, $m=M/N=N^{-1} \langle \sum _{i=1}^N s_i\rangle$. The results show that irrespective of the choice of the rescaling parameter *a*, the low temperature magnetization always coincides with the theoretical expectation, which is obtained from a brute force sampling of the partition function. Hence, *a* is here not used as a method to tune the effective temperature, as compared to the approaches mentioned above^18,19^. As discussed above, a smaller value of *a* leads to sampling of more excited states, and consequently the better the agreement with the theoretical prediction also for higher temperatures. We emphasize that a single value of the parameter *a* is sufficient to determine the low temperature behavior accurately, and the dependence on the choice of this parameter is weak, which is beneficial for applications, as no careful tuning of this degree of freedom is required.

In essence, we can consider the (imperfect) quantum annealing process as a way to find a representative set of states in the phase space which contribute strongest to the partition function from statistical mechanics due to their high Boltzmann weight. These selected configurations are used to estimate thermodynamic properties. This strategy, compared to conventional Boltzmann sampling approaches, is illustrated in Fig. 1b. The simple and robust concept is to identify potentially relevant low energy states, with no weighting according to the probability of appearance during the readout process. Instead, the proper Boltzmann weighting is then done in the approximated calculation of expectation values and the partition function, using directly the desired temperature.

### 1D Ising model

To investigate the performance of the approach also for larger systems, we consider the 1D Ising model, as in this case an analytical solution is known and allows also for comparisons in situations, where a brute force sampling of the phase space is no longer feasible. Moreover, the example differs from the previous one by having a sparse interaction matrix $J_{ij}$ and the existence of degenerate states. Therefore, these two cases cover a wide range of typical situations.

Explicitly, we use a one-dimensional Ising model with nearest neighbor ferromagnetic coupling to illustrate the calculation of thermodynamic properties using the set of states *S* sampled in analogy to the demonstration above. The model is described by the Hamiltonian ($J<0$)

4 $$\begin{aligned} H = J\sum _{k=1}^N s_k s_{k+1} + B \sum _{k=1}^N s_k \end{aligned}$$

with periodic boundary conditions ($s_{N+1}=s_1$), and has a simple analytical solution also for finite values of *N*, which serves as benchmark for the procedure. In fact, the canonical partition function is^40^

5 $$\begin{aligned} Z =\sum _{\textrm{states}} \exp (-\beta H) = \lambda _+^N + \lambda _-^N \end{aligned}$$

with the eigenvalues

6 $$\begin{aligned} \lambda _\pm = \exp (-\beta J) \left[ \cosh (\beta B) \pm \sqrt{\sinh ^2(\beta B) + \exp (4\beta J)} \right] , \end{aligned}$$

from which e.g. the Helmholtz free energy $F=-kT \ln Z$ and the magnetization per spin $m=M/N=(\partial F/\partial B)/N$ can be calculated.

**Figure 2:**
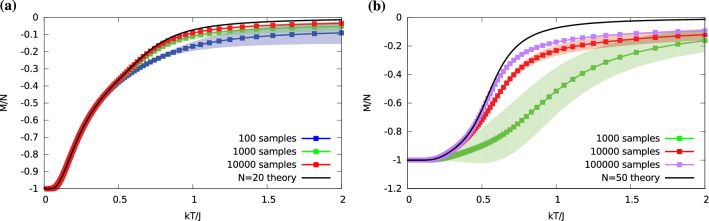
Magnetization of the 1D Ising model with periodic boundary conditions. The graphs show the magnetization per spin as function of temperature for (**a**) $N=20$ and (**b**) $N=50$ spin systems, comparing the exact analytical solution with the results from the quantum annealer. In the low temperature regime there is a perfect match, which becomes worse for higher values of *kT*/*J*. In the high temperature limit, where we expect the average magnetization to vanish, the annealer prediction saturates at finite values, as high energy states are not sampled properly. An increase of the number of annealing cycles leads to more accurate predictions, which is further enhanced by spin flip reversals. The parameters for coupling constants are $B/J=0.01$. Ten spin reversal transformations are considered for each sampling, while for 100, 000 samples in the $N=50$ system 1, 000 spin flip transformations are used. The shading illustrates the error bars of the calculations, as estimated from repeated simulations (see "Methods" section).

Again, the comparison between the exact solution and the QA sampling shows an excellent agreement of the magnetization for low temperatures, as shown in Fig. 2 for $N=20$ and $N=50$ spin systems.

For low temperatures the quantum annealing sampling is indeed in perfect agreement with the exact solution, already even for a low number of sampled configurations, which are typically generated anyway for QA applications. For higher temperatures deviations are visible, and the estimated magnetization saturates at unphysical finite values. This is an expected result, as for high temperatures all states contribute to the partition function, and then the pre-selection advantage by the QA is lost. The deviations decrease with increasing number of samples and increase with higher numbers of spins.

**Figure 3:**
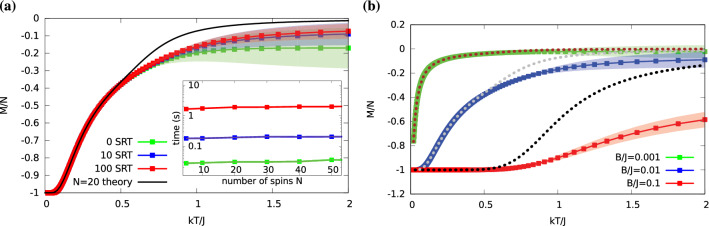
Influence of different number of spin-reversal gauge transforms and varying magnetic fields. ( **a**) Different amounts of spin-reversal transformations (SRT) during the annealing change the sampling outcomes. For QA of the N=20 spin system with $B/J=0.01$ and altogether 100 samples no, 10 and 100 SRT are used. A higher fraction of spin flips leads to more accurate predictions of the magnetization, as compared to the analytical solution (black curve). The inset shows the increasing computational demand of additional SRT. On the vertical axis, the measured QPU access time including corresponding overhead is shown as function of the number of spins in a logarithmic representation. ( **b**) Magnetization as function of temperature for different external magnetic fields *B*. The dotted lines show the exact analytical solution for the corresponding magnetic fields.

It is known that slight asymmetries in the quantum annealer can lead to favoring of specific spin alignments, and therefore spin reversal transformations, which change signs of the coupling constants without changing the physical results (see "Methods" section), can be beneficial^41^. We indeed observe a better agreement with the theoretical prediction if this feature is used. Figure 3a shows the influence of different number of spin reversal transforms on resulting magnetization and computational demand.

An increasing number of transforms lead to better results, compared to the exact analytical solution at the expense of an increase of the needed annealing time. However, quantum annealing sampling needs only a fraction of time compared to other algorithms, and therefore this increase will not be critical for many applications.

An additional analysis of the magnetic field term of the Ising Hamiltonian in Fig. 3b shows the expected alignment of spins for varying external magnetic field *B*. All curves show the expected low temperature agreement with theory, depicted as dotted lines. Surprisingly, for low magnetic fields, where the asymmetry between spin up and down configurations is lower, and where usually thermodynamic sampling is most difficult, the best agreement between simulation and theoretical prediction is reached even for elevated temperatures.

The D-Wave frontend Leap also provides functionality to influence the transformation from the transverse to the desired Hamiltonian. Usually, the associated parameters can be used to obtain the true ground state in a more reliable way. In this spirit, also reverse annealing^42^ is useful for a successful ground state search and consequently to suppress the appearance of excited states. In general, a pause in the annealing process leads to a interruption of the quantum fluctuations induced by the transverse field and allows for thermal relaxations^43^. Here, we have checked whether these features can also be used to achieve the opposite goal of the usual improved global minimization, namely a better sampling of low energy states above the ground state. It turns out that a pause in the annealing procedure has only minor influence on the expectation value of the magnetization, whereas quenching improves the chance of finding low energy states. Therefore, we have modified the annealing schedule up to the maximum possible duration of $2000\,\upmu$s, including a quench step. With such a customized annealing schedule, a better prediction of the magnetization at low temperatures is found already for just 100 samples in an $N=50$ spin system, compared to the standard schedule. Therefore, a change of the annealing protocol can lead to additional improvements for the thermodynamic predictions, although typically the effect is less pronounced than the use of the spin reversal transformations mentioned above, and therefore the standard 20 $\upmu$s annealing schedule is used for all shown plots.

**Figure 4:**
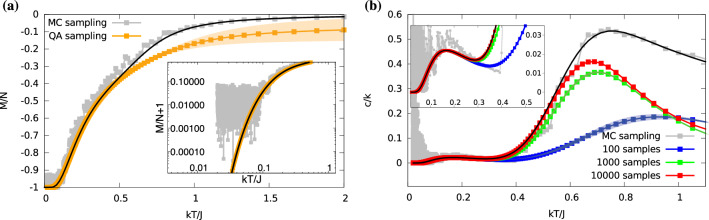
Comparison to Metropolis Monte Carlo sampling. (**a**) Comparison of the analytical theory, Metropolis Monte Carlo sampling and quantum annealing sampling for the 1D Ising model with $N=20$ spins and $B/J=0.01$. Whereas the Metropolis algorithm performs well in the high temperature regime, the results get noisy for low temperatures due to the high rejection rate of proposed states. Notice that the used $10^8$ random trial configurations correspond to more than the $2^N$ configurations of the phase space, while only 100 samples with 10 spin flip transformations are necessary for the quantum annealing sampling. The exact analytical solution is depicted as black solid line. The inset shows the magnification of the low temperature regime using a logarithmic representation of $M/N+1$. (**b**) Heat capacity per spin for the same parameter set as in panel (**a**). The plot compares the analytical solution (black curve) to the Metropolis MC sampling (grey curve) and QA predictions for different number of reads. The inset shows the magnification of the low temperature regime.

We compare the preceding QA results to conventional MC sampling using the Metropolis algorithm (see "Methods" section). For each temperature, a separate Markov chain is generated for the sampling. The comparison of both approaches is shown in Fig. 4 for the magnetization *m* and the heat capacity per spin, $c=k\beta ^2(\langle H^2\rangle - \langle H\rangle ^2)/N$. The generic and frequently used Metropolis MC approach suffers from low acceptance rates for proposed configurations at low temperatures, and therefore an accurate sampling in this regime is difficult. Exactly in this low temperature regime the quantum annealer approach plays its strength as it accesses directly the low energy configurations, which give the highest contribution to the partition function. The same set of generated configurations is used for all temperatures, like for multicanonical sampling techniques. We note that for the Monte Carlo sampling typically many more samples are necessary than for QA to get comparable results in the low temperature regime, and this number increases significantly for larger spin systems. Due to the focus on the QA approach, we refrain from further MC code optimization, Wang-Landau sampling and a comparison to other algorithms, which can perform well also for low temperatures. The presently suggested approach can become most relevant in situations, where QA is anyway used for identifying ground state configurations, as then at almost no additional computational cost also thermodynamic properties can be obtained. Altogether, we find that the different approaches complement each other very well, in particular since the QA approach is most suitable in the low temperature regime.

## Discussion

Quantum annealing is an efficient approach to determine global minima of complex energy landscapes, which are described by Ising Hamiltonians or, equivalently, quadratic unconstrained binary optimization (QUBO) problems. Due to machine imperfections and the stochastic nature of quantum annealing, typically several repetitions of the annealing process have to be performed, in order to find reliably the true ground state(s). Whereas for many applications the excited, higher energy states are ignored in the end, we have demonstrated here that they can be used for an efficient Monte Carlo Boltzmann sampling to obtain thermodynamic properties above absolute zero. These additional results are obtained essentially for free or at low computational cost, since in the low temperature regime often only very few configurations with energies slightly above the ground state are required to predict the low temperature thermodynamics.

The shown simulations do not exhibit artifacts which could indicate an improper sampling of energetically equivalent states, although such configurations exist in particular in the considered Ising model already due to translation invariance. In principle, e.g. an exponential bias of degenerate ground or low energy state configurations^20^ could be expected to lead to inappropriate low temperature predictions, but the current results do not show such discrepancies. We believe that the robustness of the approach relies on the fact that it is sufficient to identify each low energy configuration *x* once to be included into the set *S*, and therefore unfair sampling artifacts are screened if the number of reads is high enough. It is conceivable that for larger system sizes *N* such effects become more pronounced, and we observe that for sizes $N\approx 80$ on the used machine (Advantage system 4.1 with Pegasus topology) indeed more reads are required to obtain reliable results, and then the required computing time increases significantly. A good indication for such a need of more reads is that the identified low energy states have only a low number of realizations, as then the chances for missing relevant states is increased. In general, we note that the considered examples are not adjusted to the machine topology, and we therefore do not expect a strong machine type dependence of the prediction quality.

Let us briefly discuss limitations of the proposed sampling approach: First, the system sizes, which can be considered with QA, are currently limited due to the available machine sizes. However, the development of quantum annealing is still at its beginning and progressing fast, hence it can be expected that in the future faster and highly connected machines, especially with a higher number of accessible qubits, will be available, which will allow to study also models in higher dimensions with first and second order phase transitions also at finite temperatures. In the meantime, also the use of hybrid approaches^44^, which combine QA and classical minimization methods, may turn out to be useful also for finite temperature sampling. Such approaches are in general already provided by D-Wave, but they currently require manual repeated sampling due to the lower efficiency compared to pure QA. Also, overriding the automatic coupling constant renormalization is not possible, as the focus of the approach is to find most efficiently the true ground state. Nevertheless, the use of hybrid methods allows to study significantly larger system sizes than with pure QA^9^.

The second limitation is that QA requires to express the problem in terms of an Ising or QUBO formulation. We emphasize that the 1D Ising model was mainly used here to have an exact solution for benchmarking the results. Nevertheless, the methodology is applicable also to other problems where thermal excitations can play a role, e.g. for (weak) coherency strains in microstructures with long range elastic interactions^9^. In general, the Ising or QUBO limitation can actually be less severe as it may appear. To illustrate this, let us consider a simple three state system, $k=1,2,3$ with discrete states $x_k$ and energy levels $E_k$. This case can be represented through the Ising Hamiltonian $H=\sum _{i<j}^N J_{ij}s_is_j+\sum _i^Nh_is_i$ with $N=2$ spins. The three parameters $J_{12}$, $h_1$ and $h_2$ can be uniquely determined via a linear system of equations from the given energy values $E_k$ by identifying the states $x_k$ with spin pair configurations $(s_1, s_2)$. As the Ising model leads to $2^N$ configurations, there is one undesired state in this example, which can simply be omitted in the calculation of the thermodynamic properties. This way illustrates how the approach for obtaining low temperature data can be extended to general problems beyond the Ising or QUBO model.

Finally, it is not a priori clear up to which temperature the QA approach can deliver quantitative results. As demonstrated in this work, the use of more samples can improve the results, and a convergence study could be performed to extrapolate to the limit of infinite sample sizes. However, in practise such an approach will probably less useful, as it effectively leads to a sampling of the entire phase space, and then conventional approaches can be used more efficiently. Therefore, we believe that the QA sampling approach will be most useful to complement classical methods like Metropolis or Wang-Landau sampling, and will play its strengths in the low temperature limit at low computational overhead, where the other approaches are less suitable, in particular if anyway QA minimization is employed.

## Methods

### Quantum annealing

Like general purpose quantum computers, quantum annealers use qubits to process and store information, physically realized via superconducting loops, which represents different spin states via clockwise or anticlockwise circulating currents^45^. The interaction of these superconducting loops with external flux biases allows the construction of an energy landscape, where energy difference and barrier height are controlled via these fluxes^45^. At the start of the computation, the system is initialized in the ground state of a known Hamiltonian $H_0\sim -\sum _i \sigma _i^x$ with Pauli matrices $\sigma _i$, i.e. a strong transverse magnetic field^46,47^. During the annealing process, the Hamiltonian is turned into the desired one based on an Ising model^48^ $H_p = \sum _ih_is_i+\sum _{i<j}J_{ij}s_is_j$ with spin states $s_i=\pm 1$, bias $h_i$ and couplings $J_{ij}$ between spins $s_i$ and $s_j$, for which an energetic minimum is sought, $\min _{\{s_i=\pm 1\}} H_p$.

The annealing process follows the time dependence^49^ $H(s)=\frac{1}{2}A(s) H_0 + \frac{1}{2} B(s) H_p$ with normalized anneal parameter $s\in [0,1]$ and annealing evolution functions *A*(*s*) and *B*(*s*). For $s=0$, $A(0)\gg B(0)$, the initial, well known ground state is present, while for $s=1$, $A(1)\ll B(1)$, the system is expressed through the desired problem Hamiltonian^43^. In a standard annealing schedule the annealing parameter increases linearly, where varying this curve via pauses and quenches leads to a freezing of the system at an intermediate point with excited energy states^50^. This allows the sampling of the quantum Boltzmann distribution and a comparison towards classical estimators shows performance advantages of the quantum annealer for increasing system sizes^50^. Also, reverse annealing is possible, where qubits are initialized in a classical state and local minima are then searched around this state^51^.

The Hamiltonians $H_0$ and $H_p$ do not commute^48^, and the time of the initial Hamiltonian to adopt the low energy state is sufficiently large to ensure the validity of the adiabatic theorem of quantum mechanics^52^, which states that a system remains in its eigenstate, if changes occur adiabatically. Nevertheless, the machines are not perfect and do not always adopt the corresponding low energy state of the system. Therefore, also higher energy states are found, which differ from the ground state, especially if energetically close low energy states exist, a suitable number of repetitions is made and the annealing process is repeated according to a specified number of reads.

In our work the application of spin flip reversals is beneficial and improves the sampling further. This feature reflects that the machine is technically not absolutely invariant under an inversion of spins due to slight asymmetries. To overcome an artificial bias, the annealer can automatically transform the couplings according to $h_i\rightarrow h_i g_i$ and $J_{ij} \rightarrow J_{ij} g_i g_j$ with random gauges $g_i\in \{-1,+1\}$, which leave the physical problem invariant.

We use the D-Wave framework Leap^53^, as it allows to directly formulate the problem in terms of an Ising Hamiltonian. The standard embedding composite *EmbeddingComposite*, which automatically minor-embeds^54^ a problem into a sampler, is used in this work. Depending on the problem size, the given number of reads (samples) is distributed over several backend calls due to time limits of individual calls.

All quantum annealing calculations are repeated $n=10$ times to determine the standard deviation $\sigma = \pm \sqrt{\frac{1}{n-1} \sum ^n_{i=1}(x_i-\bar{x})^2}$, which is presented as shaded area in the plots. Here, *n* refers to the number of experiment repetitions, and for each of them, the given total number of reads is used.

### Metropolis Monte Carlo

Starting point of the Metropolis Monte Carlo sampling is the generation of random spin configurations. In each iteration all spins are flipped and this new configuration is accepted, if the energy is lower than the previous one, i.e. $\Delta E<0$. If the new energy is higher, the configuration is accepted with a probability given by the Boltzmann factor $\textrm{exp}(-\Delta E / k T)$. For each configuration in particular the magnetization $M=\sum _{i=1}^N s_i$ is calculated and averaged along the generated trajectory. These calculations are repeated for each temperature.

## Data Availability

Data that was obtained during this project will be made available by the corresponding author upon request.
